# Supplementary material for: The Impact of the COVID-19 Pandemic on the Number of Cancer Patients and Radiotherapy Procedures in the Warmia and Masuria Voivodeship
Source: Curr Oncol. 2023 Jan 11;30(1):1010–9. doi: 10.3390/curroncol30010077 (PMC9858600; doi:10.3390/curroncol30010077)
Supplement: Supplementary file 1 [file curroncol-30-00077-s001.zip › curroncol-2107910-supplementary.pdf]

Table S1. Distribution of RT procedures and patients due to cancer localization.

	**RT procedures** ***p*****<0.001**				**Patients** ***p*****<0.001**			
	**OLSZTYN**		**ELBLĄG**		**OLSZTYN**		**ELBLĄG**	
Cancer localization	n	(%)	n	(%)	n	(%)	n	(%)
Head and neck	263	8.4	187	7.4	246	8.6	181	8.7
Upper digestive system	95	3.0	82	3.2	83	2.9	72	3.4
Lower digestive system	291	9.2	158	6.2	272	9.6	137	6.6
Respiratory system	798	25.3	783	30.9	684	24.0	578	27.7
Breast	731	23.2	501	19.8	670	23.5	426	20.4
Gynecological	215	6.8	127	5.0	203	7.1	121	5.8
Prostate	512	16.3	453	17.9	478	16.8	398	19.0
Urinary system (without prostate)	129	4.1	135	5.3	103	3.6	87	4.2
CNS	74	2.4	59	2.3	73	2.6	59	2.8
FPI	40	1.3	46	1.8	36	1.3	31	1.5

Table S2. The number of radiotherapy procedures and patients treated with radiation therapy in the Warmia-Masuria voivodeship due to different cancer localization.

	**RT procedures**							**Patients**						
	**Before pandemic**		**During pandemic**					**Before pandemic**		**During pandemic**				
	n	(%)	n	(%)	change	(%)	p	n	(%)	n	(%)	change	(%)	p
Head and neck														
palliative RT	102	42	68	33	-34	(33)	0.04	95	42	59	30	-36	(38)	0.01
radical RT	140	58	140	67	0	(0)		133	58	140	70	7	(5)	
total	242		208		-34	(14)		228		199		-29	(13)	
Upper digestive system														
palliative RT	34	43	49	62	15	(44)	0.36	28	39	40	48	12	(43)	0.31
radical RT	45	57	49	62	4	(9)		43	61	44	52	1	(2)	
total	79		98		19	(24)		71		84		13	(18)	
Lower digestive system														
palliative RT	91	38	71	34	-20	(22)	0.43	77	35	60	31	-17	(22)	0.40
radical RT	150	62	137	66	-13	(9)		141	65	131	69	-10	(7)	
total	241		208		-33	(14)		218		191		-27	(12)	
Respiratory system														
palliative RT	438	52	395	54	-43	(10)	0.50	368	55	324	55	-44	(12)	0.86
radical RT	406	48	342	46	-64	(16)		306	45	264	45	-42	(14)	
total	844		737		-107	(13)		674		588		-86	(13)	
Breast														
palliative RT	223	34	164	28	-59	(26)	0.02	175	31	133	25	-42	(24)	0.06
radical RT	428	66	417	72	-11	(3)		397	69	391	75	-6	(2)	
total	651		581		-70	(11)		572		524		-48	(8)	
Gynecological														
palliative RT	84	46	81	51	-3	(4)	0.41	76	44	75	49	-1	(1)	0.41
radical RT	98	54	79	49	-19	(19)		95	56	78	51	-17	(18)	
total	182		160		-22	(12)		171		153		-18	(11)	
Prostate														
palliative RT	157	32	148	32	-9	(6)	0.99	140	30	116	28	-24	(17)	0.41
radical RT	340	68	320	68	-20	(6)		320	70	300	72	-20	(6)	
total	497		468		-29	(6)		460		416		-44	(10)	
Urinary system (without prostate)														
palliative RT	89	73	103	73	14	(16)	0.94	70	74	74	78	4	(6)	0.50
radical RT	33	27	39	27	6	(18)		25	26	21	22	-4	(16)	
total	122		142		20	(16)		95		95		0	(0)	
CNS														
palliative RT	18	27	20	30	2	(11)	0.66	18	27	19	29	1	(6)	0.76
radical RT	49	73	46	70	-3	(6)		49	73	46	71	-3	(6)	
total	67		66		-1	(1)		67		65		-2	(3)	
FPI														
palliative RT	40	78	23	66	-17	(43)	0.19	31	82	21	72	-10	(32)	0.37
radical RT	11	22	12	34	1	(9)		7	18	8	28	1	(14)	
total	51		35		-16	(31)		38		29		-9	(24)	

Table S3. The number of radiotherapy procedures and patients treated with radiation therapy in Olsztyn due to different cancer localization.

	RT procedures							Patients						
	Before pandemic		During pandemic					Before pandemic		During pandemic				
	n	(%)	n	(%)	change	(%)	p	n	(%)	n	(%)	change	(%)	p
Head and neck														
palliative RT	48	34	37	30	-11	(23)	0.47	41	31	29	25	-12	(29)	0.29
radical RT	92	66	86	70	-6	(7)		90	69	86	75	-4	(4)	
total	140		123		-17	(12)		131		115		-16	(12)	
Upper digestive system														
palliative RT	28	54	27	63	-1	(4)	0.38	22	48	21	57	-1	(5)	0.42
radical RT	24	46	16	37	-8	(33)		24	52	16	43	-8	(33)	
total	52		43		-9	(17)		46		37		-9	(20)	
Lower digestive system														
palliative RT	45	28	43	32	-2	(4)	0.48	35	24	36	29	1	(3)	0.35
radical RT	113	72	90	68	-23	(20)		112	76	89	71	-23	(21)	
total	158		133		-25	(16)		147		125		-22	(15)	
Respiratory system														
palliative RT	265	61	227	62	-38	(14)	0.71	220	58	180	59	-40	(18)	0.87
radical RT	169	39	137	38	-32	(19)		158	42	126	41	-32	(20)	
total	434		364		-70	(16)		378		306		-72	(19)	
Breast														
palliative RT	111	29	81	23	-30	(27)	0.07	76	22	60	18	-16	(21)	0.22
radical RT	271	71	268	77	-3	(1)		267	78	267	82	0	(0)	
total	382		349		-33	(9)		343		327		-16	(5)	
Gynecological														
palliative RT	55	49	51	50	-4	(7)	0.85	49	46	46	47	-3	(6)	0.86
radical RT	58	51	51	50	-7	(12)		57	54	51	53	-6	(11)	
total	113		102		-11	(10)		106		97		-9	(8)	
Prostate														
palliative RT	71	25	47	20	-24	(34)	0.19	58	22	32	15	-26	(45)	0.06
radical RT	210	75	184	80	-26	(12)		207	78	181	85	-26	(13)	
total	281		231		-50	(18)		265		213		-52	(20)	
Urinary system (without prostate)														
palliative RT	58	89	57	89	-1	(2)	0.98	45	87	44	86	-1	(2)	0.97
radical RT	7	11	7	11	0	(0)		7	13	7	14	0	(0)	
total	65		64		-1	(2)		52		51		-1	(2)	
CNS														
palliative RT	5	13	8	24	3	(60)	0.21	5	13	7	21	2	(40)	0.32
radical RT	35	88	26	76	-9	(26)		35	88	26	79	-9	(26)	
total	40		34		-6	(15)		40		33		-7	(18)	
FPI														
palliative RT	19	79	16	100	-3	(16)	0.05	16	76	15	100	-1	(6)	0.04
radical RT	5	21	0	0	-5	(100)		5	24	0	0	-5	(100)	
total	24		16		-8	(33)		21		15		-6	(29)	

Table S4. The number of radiotherapy procedures and patients treated with radiation therapy in Elbląg due to different cancer localization.

	**RT procedures**							**Patients**						
	**Before pandemic**		**During pandemic**					**Before pandemic**		**During pandemic**				
	n	(%)	n	(%)	change	(%)	p	n	(%)	n	(%)	change	(%)	p
Head and neck														
palliative RT	54	53	31	36	-23	(43)	0.02	54	56	30	36	-24	(44)	0.007
radical RT	48	47	54	64	6	(13)		43	44	54	64	11	(26)	
total	102		85		-17	(17)		97		84		-13	(13)	
Upper digestive system														
palliative RT	6	22	22	40	16	(267)	0.11	6	24	19	40	13	(217)	0.16
radical RT	21	78	33	60	12	(57)		19	76	28	60	9	(47)	
total	27		55		28	(104)		25		47		22	(88)	
Lower digestive system														
palliative RT	46	55	28	37	-18	(39)	0.02	42	59	24	36	-18	(43)	0.008
radical RT	37	45	47	63	10	(27)		29	41	42	64	13	(45)	
total	83		75		-8	(10)		71		66		-5	(7)	
Respiratory system														
palliative RT	173	42	168	45	-5	(3)	0.42	148	50	144	51	-4	(3)	0.80
radical RT	237	58	205	55	-32	(14)		148	50	138	49	-10	(7)	
total	410		373		-37	(9)		296		282		-14	(5)	
Breast														
palliative RT	112	42	83	36	-29	(26)	0.18	99	43	73	37	-26	(26)	0.20
radical RT	157	58	149	64	-8	(5)		130	57	124	63	-6	(5)	
total	269		232		-37	(14)		229		197		-32	(14)	
Gynecological														
palliative RT	29	42	30	52	1	(3)	0.28	27	42	29	52	2	(7)	0.26
radical RT	40	58	28	48	-12	(30)		38	58	27	48	-11	(29)	
total	69		58		-11	(16)		65		56		-9	(14)	
Prostate														
palliative RT	86	40	101	43	15	(17)	0.55	82	42	84	41	2	(2)	0.89
radical RT	130	60	136	57	6	(5)		113	58	119	59	6	(5)	
total	216		237		21	(10)		195		203		8	(4)	
Urinary system (without prostate)														
palliative RT	31	54	46	59	15	(48)	0.59	25	58	30	68	5	(20)	0.33
radical RT	26	46	32	41	6	(23)		18	42	14	32	-4	(22)	
total	57		78		21	(37)		43		44		1	(2)	
CNS														
palliative RT	13	48	12	38	-1	(8)	0.41	13	48	12	38	-1	(8)	0.41
radical RT	14	52	20	63	6	(43)		14	52	20	63	6	(43)	
total	27		32		5	(19)		27		32		5	(19)	
FPI														
palliative RT	21	78	7	37	-14	(67)	0.005	15	88	6	43	-9	(60)	0.007
radical RT	6	22	12	63	6	(100)		2	12	8	57	6	(300)	
total	27		19		-8	(30)		17		14		-3	(18)	

Figure S1. Distribution of RT procedures due to cancer localization. (**A**) Head and neck (ICD-10: C00-C14; C30-C32), (**B**) Upper digestive system (ICD-10: C15; C16; C22; C24; C25; C26; D37), (**C**) Lower digestive system (ICD-10: C17-C21), (**D**) Respiratory system (ICD-10: C33-C35; C38; C45; D38), (**E**) Breast (ICD-10: C50), (**F**) Gynecological (ICD-10: C51-C54; C56-C57), (**G**) Prostate (ICD-10: C61), (**H**) Urinary system (without prostate) (ICD-10: C64; C65-C67), (**I**) CNS (ICD-10: C70-C72; D43), (**J**) FPI (ICD-10: C80).

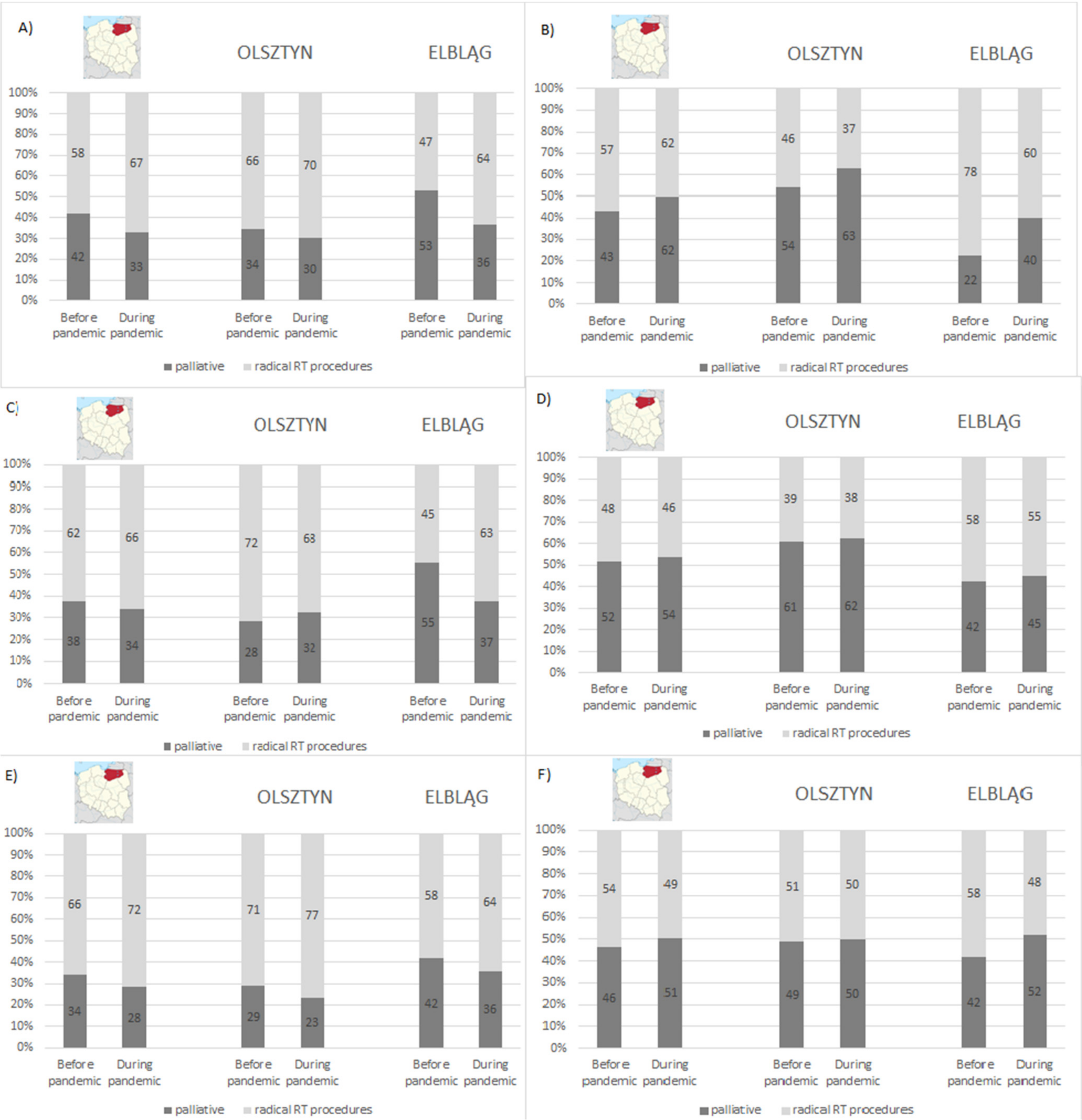

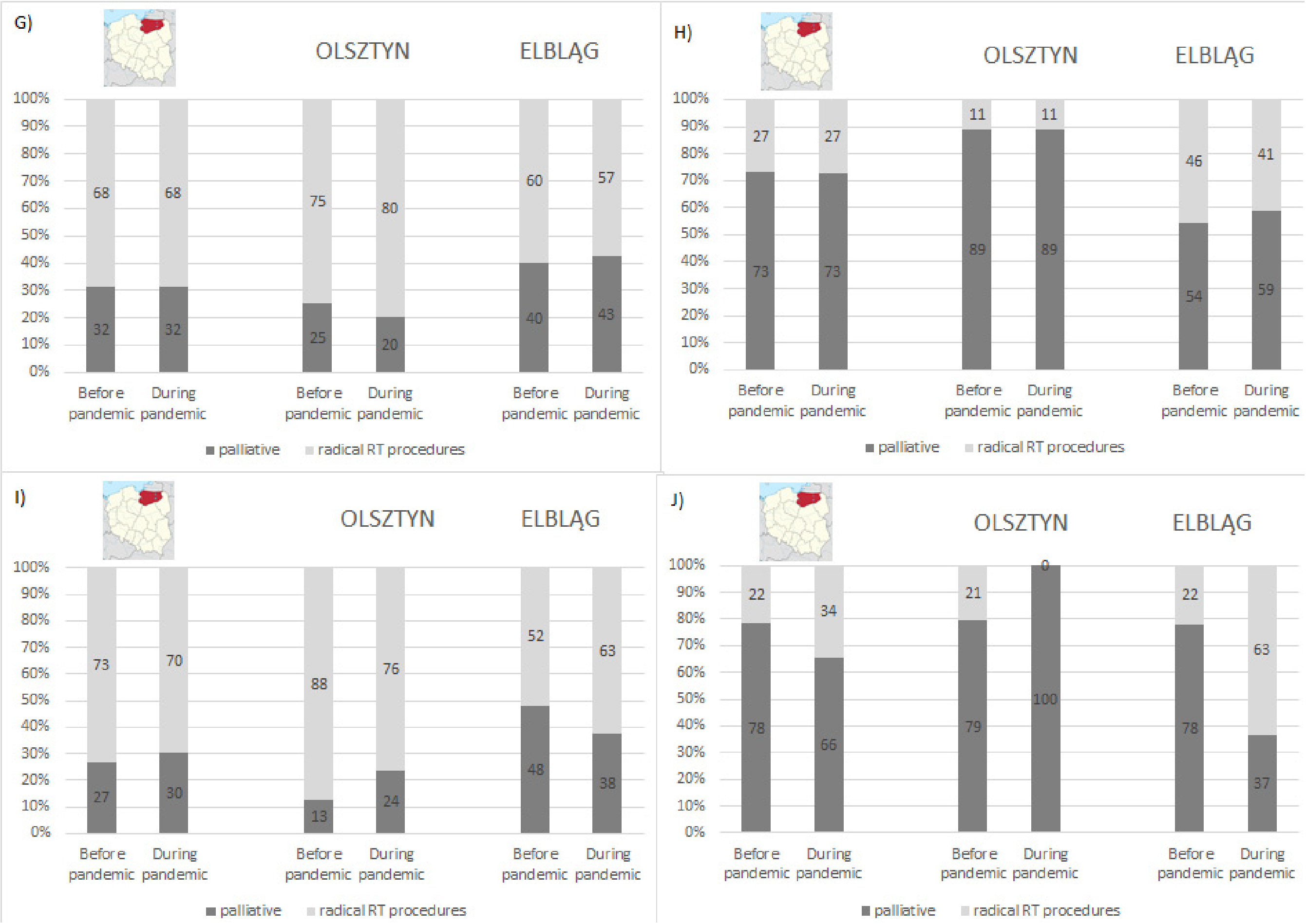
G)
OLSZTYN
ELBLĄG
100%
90%
80%
70%
60%
50%
40%
30%
20%
10%
0%
68
68
75
80
60
57
32
32
25
20
40
43
Before pandemic
During pandemic
palliative
radical RT procedures
H)
OLSZTYN
ELBLĄG
27
27
11
11
46
41
73
73
89
89
54
59
Before pandemic
During pandemic
palliative
radical RT procedures
I)
OLSZTYN
ELBLĄG
73
70
88
76
52
63
27
30
13
24
48
38
Before pandemic
During pandemic
palliative
radical RT procedures
J)
OLSZTYN
ELBLĄG
22
34
21
0
22
63
78
66
79
100
78
37
Before pandemic
During pandemic
palliative
radical RT procedures

Figure S2. Distribution of patients treated with RT due to cancer localization. (**A**) Head and neck (ICD-10: C00-C14; C30-C32), (**B**) Upper digestive system (ICD-10: C15; C16; C22; C24; C25; C26; D37), (**C**) Lower digestive system (ICD-10: C17-C21), (**D**) Respiratory system (ICD-10: C33-C35; C38; C45; D38), (**E**) Breast (ICD-10: C50), (**F**) Gynecological (ICD-10: C51-C54; C56-C57), (**G**) Prostate (ICD-10: C61), (**H**) Urinary system (without prostate) (ICD-10: C64; C65-C67), (**I**) CNS (ICD-10: C70-C72; D43), (**J**) FPI (ICD-10: C80).

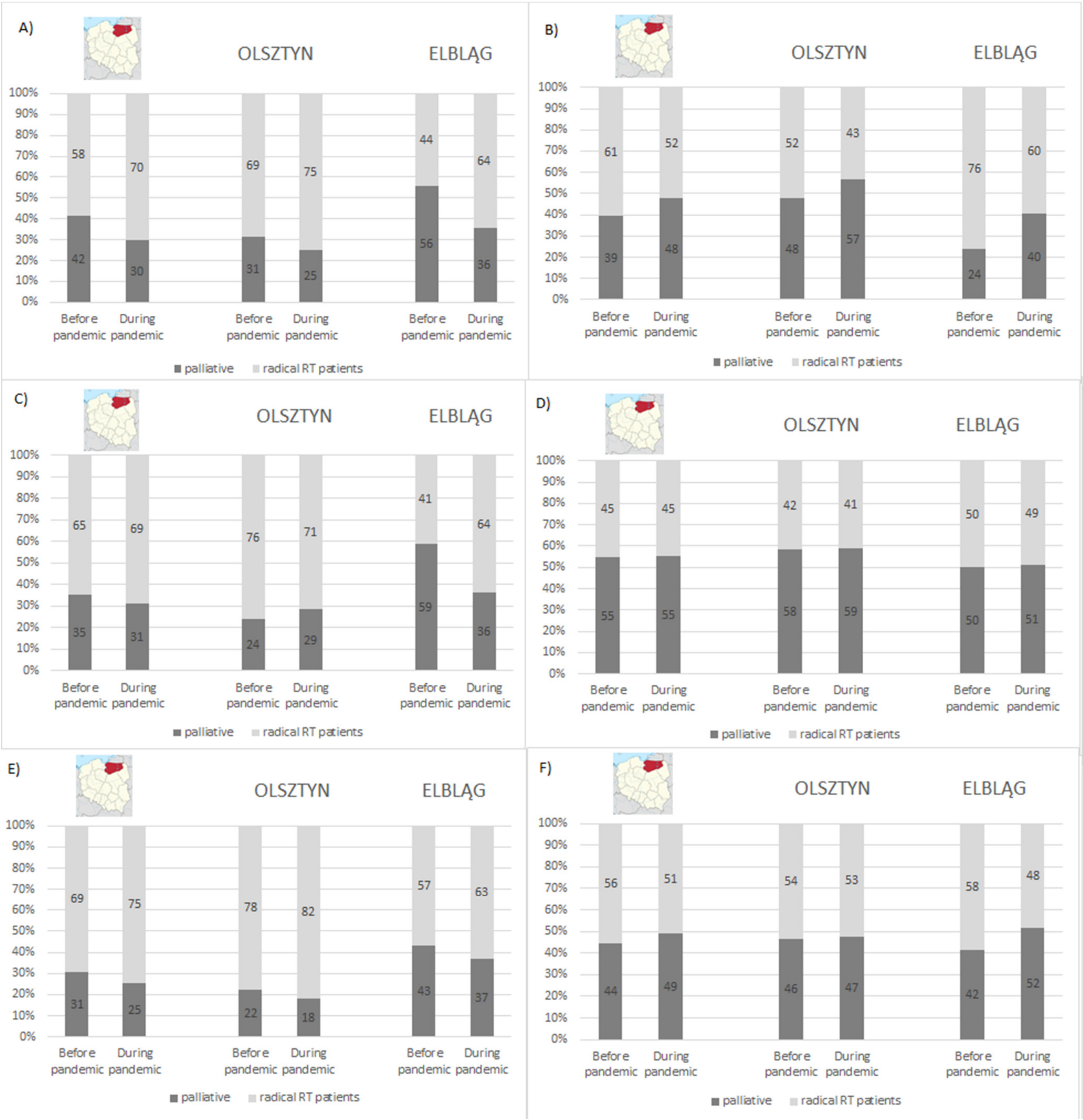

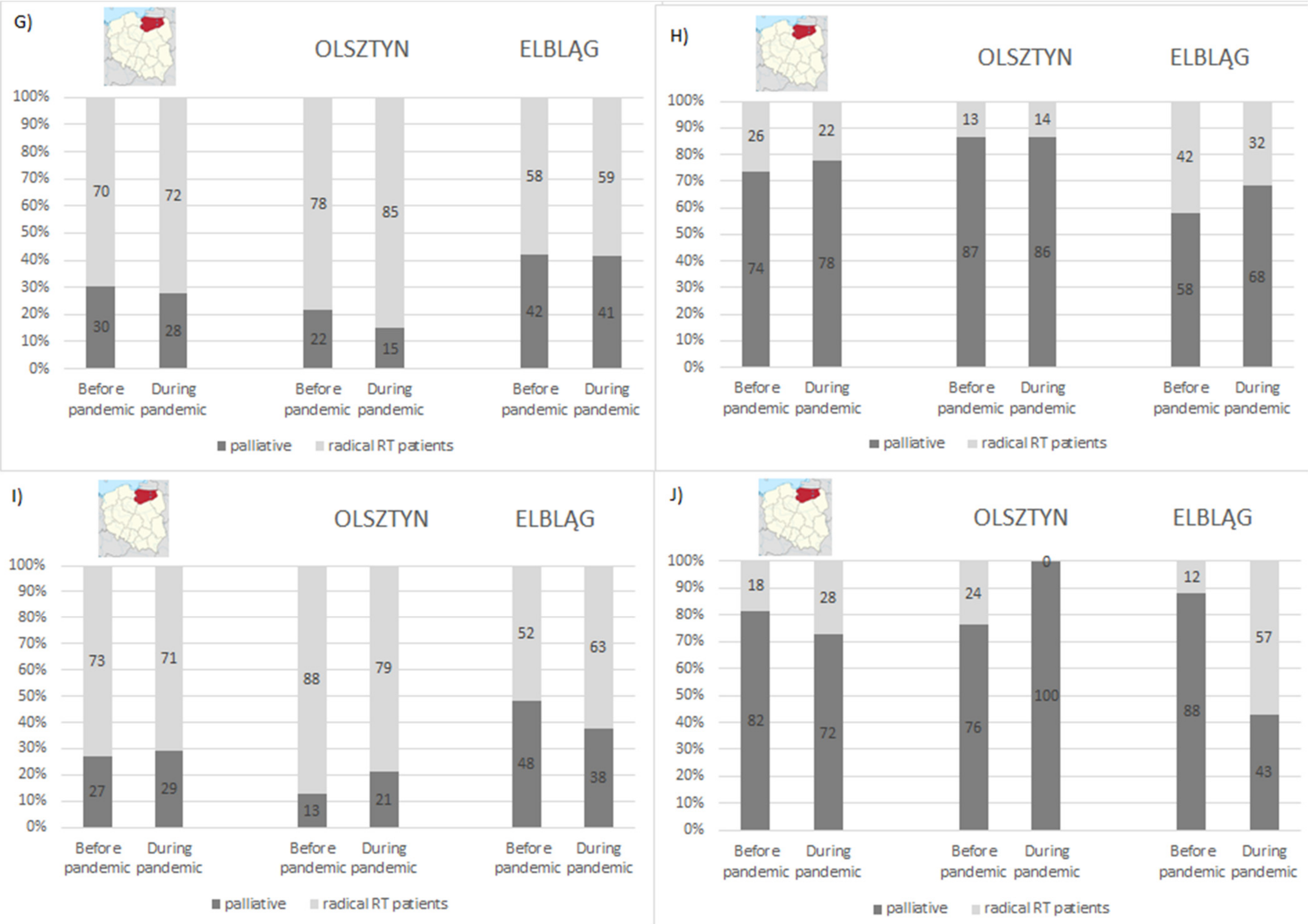

G)
OLSZTYN
ELBLĄG
100%
90%
80%
70%
60%
50%
40%
30%
20%
10%
0%
70
72
78
85
58
59
30
28
22
15
42
41
Before pandemic
During pandemic
palliative
radical RT patients
H)
OLSZTYN
ELBLĄG
26
22
13
14
42
32
74
78
87
86
58
68
Before pandemic
During pandemic
palliative
radical RT patients
I)
OLSZTYN
ELBLĄG
73
71
88
79
52
63
27
29
13
21
48
38
Before pandemic
During pandemic
palliative
radical RT patients
J)
OLSZTYN
ELBLĄG
18
28
24
0
12
57
82
72
76
100
88
43
Before pandemic
During pandemic
palliative
radical RT patients
